# Dental Anomalies and Dental Age Assessment in Treated Children with Acute Lymphoblastic Leukemia

**Published:** 2014-12-10

**Authors:** L Khojastepour, S Zareifar, M Ebrahimi

**Affiliations:** 1 Department of Oral and Maxillofacial Radiology, Shiraz University of Medial Science, Shiraz, Iran; 2 Hematology Research Center, Shiraz University of Medical Sciences, Shiraz, Iran.; 3 Faulty of Dentistry, Shiraz University of Medial Science, Shiraz, Iran.

**Keywords:** Acute Lymphoblastic Leukemia, Dental anomalies, Dental age, Chemotherapy.

## Abstract

**Background:**

This cross sectional study was performed to evaluate dental ages and incidence of dental anomalies in children treated for acute lymphoblastic leukemia (ALL).

**Methods and materials:**

A total of 25 ALL patient who passed at least 2 years of chemotherapy and 25 healthy sex and age matched children were evaluated. Dental age as well as dental anomalies in shape, size, number, and structure was recorded based on their panoramic radiographies which were taken for dental purposes.

**Results:**

The number of dental anomalies significantly increased in ALL treated children. Seven ALL cases (28%) in compression to only one (4%) in control group had at least one dental anomaly. However, there was neither statistically significant differences between the mean of dental (p=0.32) and chronologic age (p=0.12) in both groups, nor between dental age of cases and control group (p=0.62).The age at the onset of treatment as well as treatment durations has not affected dental age and the incidence of dental anomalies significantly (p<0.05).

**Conclusion:**

Chemotherapy in children results in emergence of dental anomaly. Dental age, maturity, and development process however seems to be independent from chemotherapy.

## Introduction

Cancer is one of the most frequent causes of death in children and leukemia is the most common childhood malignancy. It happens due to genetic abnormality of hematopoietic cell proliferation and differentiation (1). In fact, it is an abnormal bone marrow function. Among the types of leukemia, acute lymphoblastic leukemia (ALL) includes the highest percentage and affects the children up to the age of 14 years. Treatment of choice for hematological malignancy is through a single or the combination use of Chemotherapy, radiotherapy and bone marrow transplant. The survival rate of childhood cancer has been increased by current multimodality therapies (1-3).Treatment of ALL as the most common type of childhood malignancy is done during the odontogenesis period so the side effects of treatment choices on dental development and maturation can affect the quality of life of survived patients. The long term adverse effect of anti cancer therapy on dental maturation and development most often were considered generally and factors such as child's age at the time of diagnosis and treatment, treatment protocol, type of prescribed medications, dosage, and frequency of consumption and intervention at the appropriate time are mentioned as possible factors affecting the dental consequences. Enamel hypoplasia, Delayed eruption, shortened malformed roots, and failure to form one or more teeth are listed among the effects of anticancer therapy on odontogenesis and tooth development (2-11).

The aim of chemotherapy is destruction of tumor cells but the chemotherapy agents have not selective toxicity for tumor cells. Normal cells especially in proliferative phase also are affected by chemotherapy as a systemic treatment. Mucositis, temporary dry mouth, infection, and gingival bleeding are the most common short term side effects that are observed in oral cavity. The interference of chemotherapy agents with the cell metabolic cycle on developing dentition of children also could induce delayed dental development and dental abnormality, including hypodontia, microdontia, enamel hypoplasia, root stunting and taurodontia (1,5-7).

For a while now there have been a few researches in which the effects of chemotherapy, as the only treatment modality have been considered on odontogenesis and dental maturity. Moreover, in the case of dental maturity and dental age they have not yielded consistent result (5,6,12,13).

In this study, we compared the incidence of dental anomalies in shape, size, number, structure, and dental age of ALL patients who treated only by chemotherapy with a matched age and sex control group. The effects of treatment duration and the age at treatment onset were also investigated.

## Material and methods

This cross sectional study was performed on panoramic radiographs that were taken for dental purposes from 25 ALL patients, including nine girls and 16 boys. They were in the age range of 4-14 years and received only chemotherapy treatment for at least two years in oncology ward. ALL patients who passed radiotherapy and or bone marrow transplantation were excluded. For control group, panoramic radiographs of the 25 healthy sex and age matched cases that were taken for dental reasons were used. Systemic diseases that could affect the result of this study, such as osteomalacia rickets, ectodermal dysplasia, hypophosphatasia, and Down's syndrome considered as exclusion criteria for control group. Demographic information including patient's age, sex, age at the time of diagnosis and treatment, type of chemical agents, and chemotherapy duration, were recorded from the patients’ files. The Shiraz University of Medical Sciences Ethics Committee approved the study and informed contest were taken from the parents of participants. All the radiographs were taken with the same panoramic machine (CRANEX D SOREDEX, Finland**)**. The panoramic radiographies were assessed in ambient light conditions for dental anomaly and dental age by an oral radiologist. Dental age was determined based on three characteristic: which teeth have erupted, the amount of resorption of the root of primary teeth, and the amount of development of the permanent teeth (chronology of tooth development, permanent dentition (14).

Data were statistically analyzed using SPSS version 15 software package for windows (Chicago, IL, USA). Fisher Exact test was used to compare anomalies incidence between case and control groups. Independent t-test was used for assessing the effect of the age at the time of ALL diagnosis (treatment onset) and duration of chemotherapy treatment on the emergence of dental anomalies. The mean of dental and chronologic age in both groups were also compared with independent t-test. P-value less than 0.05 was considered statistically significant. 

## Results

The mean of chronologic age was 7.8± 3.38 and 8.2±3.6 years in case and control groups, respectively. The average age of patients at the time of diagnosis and beginning of treatment was 5.16 years. In both groups the mean dental age was more than Chronological age, but this difference was not statistically significant (Table I). Regarding the dental age, there wasn't any significant difference between cases and control group (p=0.62).

Distributions of dental anomalies in case and control groups are shown in Table 2. It was a statistically significant difference between the incidence of dental anomalies in case and control groups (p=0.02). Seven out of 25 ALL treated children (28%) had at least one dental anomaly compared with only one (4%) in control group. Taurodontism with 16 %frequency had been the most commenly found anomaly. Treatment duration and the age of treatment onset for cases with and without dental anomaly were shown in Table 3. There was neither a statistically significant difference between the treatment duration or the age at the beginning of treatment among treated ALL cases with and without dental anomaly (p=0.83 and p=0.07, respectively).

**Table I:** Comparison Between Chronological and Dental Age in ALL treated patients and healthy control groups

	**Chronological Age** **Mean ± SD**	**Dental Age** **Mean ± SD**	**P-value**
**ALL Cases**	7.8 ± 3.38	8.04 ± 3.67	0.32
**Control Group**	8.2 ± 3.60	8.52 ± 3.25	0.12

**Table II:** Prevalence of dental anomalies in ALL treated patients and control groups

**Type of detected Anomaly**	**ALL Cases** **(N=25)**	**Control Group** **(N=25)**
**Taurodontism**	4 (25%)	1 (4%)
**Spiky and short root**	2 (8%)	0 (0%)
**Missing**	1 (4%)	0 (0%)
**Total**	7 (28%)	1(4%)

**Table III:** Treatment duration and age at treatment onset in ALL treated patients with and without anomaly

	**ALL cases with Anomaly (N=7)**	**ALL cases without Anomaly (N=18)**	**P-value**
**Age at ALL Diagnosis (Year)**	4.4±2.5	7±3.3	0.07
**Treatment duration (Month)**	33.28±20.13	30.33±12.13	0.83

## Discussion

Local and systemic factors such as illness, trauma, chemotherapy or radiotherapy could possibly affect the dental development and odontogenesis process at any time prior to their complete formation. The increased survival rate of patients with childhood malignancies will increase the chance of emergence of compromising long term health problems resulting from delayed toxicities of modern therapy. Among various protocols of cancer treatment, long term effects of radiotherapy on dentition are far more discussed. Dental abnormalities also were reported in survived patient who passed chemotherapy alone or in combination with other treatment modalities. 

In the present study we found higher percentage of dental anomalies among treated ALL cases than control group which was in accordance with the previous studies. According to Goho theory, Odontogenic cell sensitivity is dependent upon the position on the cell cycle and the mitotic activity at the time of chemoradiatiotn therapy. He emphasizes that while Chemotherapy damage is related directly to the doses and repetition of the various agents, it will affects only those Odontoblasts and ameloblasts who are in susceptible phases of the cell cycle and Cells in nonproliferative, germinal stages (for example second or third permanent molars in the infant) are unaffected and should develop normally. Our result was inaccordance to Goho theory as there was no detectable anomaly in second molar (7).

Kaste et al (2) in a study reviewed the clinical records and panoramic radiographs of 423 survivors of ALL for defining the therapy-associated dental abnormalities among them. Dental anomalies, including root stunting, microdontia, hypodontia, taurodontia (enlarged pulp chambers), and over-retention of primary teeth were reported and their frequency was determined in relation to age at initiation of treatment (< or = 8 years vs. > 8 years), addition of cranial irradiation, and chemotherapeutic protocol. Root stunting, microdontia, hypodontia, taurodontia and over-retention of primary dentition were described in order. They also found more dental anomalies in patients who were ≤ 8 years old at diagnosis or who received cranial irradiation therapy than those > 8 years and those who did not receive cranial irradiation. In contrast to their result, in the present study taurodontism was the most common finding followed by spiky, short root and missing teeth (hypodontia). Also regardless of lower mean age at the time of ALL diagnosis in survived patients who developed dental anomalies (4.4 ± 2.5 compared to 7 ± 3.3) this difference was not statistically significant. It could be related to the limited survived patient who met the study criteria.

Kaste et al(3) in a multivalent analysis based on a 24-page, self reported questionnaire also identified that exposure to alkylating agents, independent of radiation exposure induced dose-associated and age-associated adverse dental outcomes in childhood cancer survivors(3). Minicucci et al observed higher frequency of dental abnormality in children who were only subjected to chemotherapy. They also found different types of dental abnormalities depending on protocols used. Hypodontia and shortened root were more common when chemotherapy and radiotherapy were used together while microdontia and delayed dental development showed higher incidence when chemotherapy was used alone (1).

Lopes and colleagues in the study conducted on 137 cases evaluated dental abnormalities in children who were submitted to antineoplastic therapy. They observed that 28% of children with chemotherapy alone or with radiation therapy had experienced an anomaly. The mean age of patients at the beginning of treatment was 5.5 years. Reported anomalies include microdontia, anodontia, taurodontia, macrodontia, blunted, and tapered roots while taurodontism was the most frequent anomaly. In this study also taurodontism has the highest frequency which is in accordance with Lopes et al study (9).

More recently Lauritano and Petruzzi (8) also evaluated the possible effects of the anti-leukaemic therapy on the dental development of fifty-two long term children survived leukaemia, aged from 8 to 15 years, including 27 females, 25 males with mean age 11.5 years who were at least 24 months in continuous complete remission. In addition, they compared the results with the matched control group. The study of the dental status was performed by a routine oral examination and panoramic radiographs. The results of this study evidence that long-term children survived leukaemia in comparison to the control group have a higher risk to develop dental caries and show a greater severity of dental anomalies including V-shaped roots, dental agenesis, microdontia, and enamel dysplasia and these are in agreement with the data reported in other studies(1, 2, 9, 10 ).

The average duration of chemotherapy was 31.8 months. The influence of treatment period on the emergence of anomaly was not significant either. It may be due to dispersion of treatment duration among them.

Dental age is dependent on dental maturity stage in growing children and in comparison to chronological age was considered as an indicator of biological maturity. Regarding this issue, we did not find any difference between dental age and chronological age of treated ALL patients and control group. This is in accordance with some and in contrast with the other studies (5,6, 12,13). Dahllöf and colleges evaluated the effect of chemotherapy on dental maturity of 44 children with hematological malignancies. They found that there is no significant difference between the chronological and dental age in children treated with chemotherapy compared to healthy controls. The result of present study is in accordance with Dahllöf and^.^ Pajari et al (13) also assessed the effect of anti- neoplasic therapy on dental maturity and tooth development and found that regardless of advanced average dental age in 38 children who received chemotherapy with or without radiation the difference was not statistically significant (13). In contrast to the present study, Vasconcelos et al (12) found advanced dental age in treated ALL patients compared to control group while Purdell-Lewis et al ^5^ reported delayed dental eruption after chemotherapy. Vasconcelos et al also found that different protocols for ALL treatment had the same effect on dental maturity (12). They used a chart created and adapted by the author, from the original chart of Demirjian’s et al( 15 )and explained that since cancer therapy is responsible for the premature closure of apices (2,13,16,17) perhaps the method proposed by Demirjian’s et al. for dental assessment was not suitable for their study. They pointed to the facts that concomitant use of other medications during treatment, the disease itself, and nutritional factors can also interfere with tooth formation. Moreover, they proposed that Demirjian’s method for dental assessment should be adapted to different populations by considering individual assessment parameter and ethnic difference (12).

## Conclusion

According to the results of this study, if it could be ignored the relatively small group of survived ALL patients with the study criteria, we could conclude that genetic and ethnic factors have more effect on dental maturity and dental age than ALL and or chemotherapy treatment. For better assessment of the effect of age at the onset of treatment and duration of chemotherapy on tooth development, it is recommended that if it is possible evaluate younger patients in a larger cohort study.
